# The association between mental healthcare professionals’ personal characteristics and their clinical lifestyle practices: a national cross-sectional study in The Netherlands

**DOI:** 10.1192/j.eurpsy.2023.2475

**Published:** 2023-12-04

**Authors:** Lisanne E.M. Koomen, Jeroen Deenik, Wiepke Cahn

**Affiliations:** 1 UMC Utrecht, dep. Psychiatry, Utrecht, The Netherlands; 2 Lister, Utrecht, The Netherlands; 3 Maastricht University, Fac. Health, Medicine and Life Sciences, Maastricht, The Netherlands; 4 GGz Centraal, dep. Research, Amersfoort, The Netherlands; 5 Altrecht Mental Health Institute, Utrecht, The Netherlands

**Keywords:** cross-sectional study, implementation, lifestyle psychiatry, mental health, physical health

## Abstract

**Background:**

Lifestyle interventions are important to improve the mental and physical health outcomes of people with mental illness. However, referring patients to lifestyle interventions is still not a common practice for mental healthcare professionals (MHCPs) and their own lifestyle habits may impact this. The aim of this study was to investigate MHCPs’ personal lifestyle habits, their lifestyle history and referral practices, and if these are associated with their lifestyle habits, gender, and profession.

**Methods:**

In this cross-sectional study, an online questionnaire was distributed across relevant MHCP’s in The Netherlands. Ordinal regression analyses on lifestyle habits, gender, profession, and lifestyle history and referral practices were conducted.

**Results:**

A total of the 1,607 included MHCPs, 87.6% finds that lifestyle should be part of every psychiatric treatment, but depending on which lifestyle factor, 55.1–84.0% take a lifestyle history, 29.7–41.1% refer to interventions, and less than half (44.2%) of smoking patients are advised to quit. MHCPs who find their lifestyle important, who are physically more active, females, and MHCPs with a nursing background take more lifestyle histories and refer more often. Compared to current smokers, MHCPs who never or formerly smoked have higher odds (2.64 and 3.40, respectively, *p* < 0.001) to advice patients to quit smoking.

**Conclusions:**

This study indicates that MHCPs’ personal lifestyle habits, gender, and profession affect their clinical lifestyle practices, and thereby the translation of compelling evidence on lifestyle psychiatry to improved healthcare for patients.

## Introduction

People with mental illness often suffer from long-lasting psychiatric symptoms, despite treatment with medication or psychotherapy, resulting in lower social functioning and lower quality of life [1, 2]. They also experience impaired physical health since they have a 1.4–2 times higher risk to develop cardiometabolic diseases and have a lower life expectancy, which is partly caused by unhealthy lifestyle habits [3–5]. People with mental illness are less physical active, tend to smoke more, and eat less healthy [5]. Interventions aiming to improve lifestyle habits, for example, increasing physical activity and smoking cessation, can improve mental and physical health across a range of diagnoses [5, 6]. Therefore, international guidelines, including from the World Health Organization and the European Psychiatric Association advice lifestyle interventions in the treatment for mental illness [7–10]. For mild–moderate depression, physical activity interventions are even recommended as first-line treatment [7], and a meta-review on lifestyle psychiatry stated that physical activity interventions can be used as prevention and treatment for mental illness [6]. Moreover, The Lancet Psychiatry commission on protecting physical health in people with mental illness advised that lifestyle interventions should be available from patient’s first presentation to protect the physical health of people with mental illness [5].

However, it is still not common practice to refer patients with mental illness to lifestyle interventions to improve their disadvantaged health status. An Australian study found that 70% of the mental healthcare professionals (MHCPs) prescribed exercise daily or weekly for depression, anxiety, or stress, but only 11% for schizophrenia or bipolar disorder [11]. A Dutch study found that 80% of referring MHCPs had ever referred a patient to an exercise program. Some studies suggest that HCPs’ own lifestyle habits impact their clinical lifestyle practices [12–20]. The Dutch study found that referral to physical activity interventions was associated with MHCPs’ physical activity levels [20]. These findings are in line with previous research on lifestyle habits of the HCP and their counsel and referral practices in general healthcare. HCPs who practice healthy lifestyle habits are more likely to counsel on these lifestyle habits and non-smoking HCPs are more likely to advice their patients to quit smoking [12–19].

Although previous studies investigated the personal physical activity habits of psychiatrists and their referral practices to physical activity interventions [11, 20], it is unknown whether such associations also apply to a broader spectrum of lifestyle habits and referring professions in mental healthcare. Therefore, the aim of this study was to identify MHCPs’ lifestyle habits and their clinical practices on taking a lifestyle history, and referral regarding physical activity, dietary habits, sleep, tobacco, and alcohol use, and to investigate whether these practices are associated with their personal lifestyle habits.

## Methods

### Study design

This cross-sectional study was conducted between May 2022 and January 2023. All mental healthcare institutions, psychiatry departments of hospitals, organizations for nurse specialists, and organizations for independent working psychiatrists in The Netherlands were asked to distribute the link to the online questionnaire to referring MHCPs. In addition, invitations to the online questionnaire were distributed at the Dutch Association for Psychiatry (NVvP) 2022 congress, which is the main psychiatry congress in The Netherlands. To minimize selection bias, the invitation to the online questionnaire invited referring MHCPs to give their opinion about lifestyle psychiatry, also when they believed healthy lifestyle should not play a role in psychiatry.

### Participants

We included the following groups of referring MHCPs based on informed consent: residents in psychiatry, nurse specialists working at a general practitioner practice (GP-based nurse specialists), specialized nurses and physician assistants (category referring MHCP with a nursing background), healthcare psychologists and remedial educationalists (category referring MHCP with scientific background), clinical psychologists and psychiatrists. Although the questionnaire was aimed for referring MHCPs, also MHCPs who cannot refer patients filled in the questionnaire. They were divided into the categories psychologists and “other.” The division of profession groups was based on educational level and clinical responsibilities. Participants were included in the study when they at least completed the first two parts of the questionnaire.

Under Dutch law, medical ethical approval is not required for non-medical research with anonymous data. The rules of conduct as outlined in the Declaration of Helsinki of 1975, as revised in 2008, were followed, and participants were asked for written consent.

### Questionnaire

The questionnaire consisted of three parts. Participants were first asked about age, gender, height, weight, profession, work setting, main patient population they work with, and if lifestyle interventions were available within their work setting.

Then, questions about their own lifestyle were asked. Participants were asked how important their lifestyle is on a scale from 0 to 10. Questions on tobacco use, drinking habits, and drug use were based on the FANTASTIC instrument [21]. Questions regarding physical activity were based on international physical activity guidelines, namely ≥2x bone and muscle strengthening exercises per week, and ≥150 min moderate to vigorous physical activity (MVPA) per week [22]. For analysis, we calculated if participants adhered to these guidelines. Questions on eating pattern were based on the FANTASTIC instrument and WHO steps [21, 23]. Participants were asked on sleep satisfaction, and stress related to their work and their private life on a scale from 0 to 10.

The third part of the questionnaire consisted of questions on lifestyle within psychiatric treatment. Participants were first asked if lifestyle should be part of psychiatric treatment, and who is responsible for the lifestyle treatment of a psychiatric patient. Then, participants were questioned what proportion of their patients they ask about: physical activity, dietary habits, sleep, tobacco, and alcohol use. What proportion of their patients they advise to quit smoking if they smoke, and what proportion of their patients they refer to: lifestyle interventions to improve mental health, lifestyle interventions to improve physical health, and insomnia cognitive behavioral therapy (CBT). These questions were stated as follows: *What proportion of your patients do you refer to “an intervention aimed at improving or maintaining someone’s lifestyle” to improve their mental health, if you believe there is something to gain for the patient in terms of lifestyle?* Answer options were: 0%, 0–10%, 10–20%, 20–30%, 30–40%, 40–50%, 50–60%, 60–70%, 70–80%, 80–90%, 90–100%, and not applicable. For analysis, we recoded these answer options into mean percentages.

See the Supplementary Material for the full questionnaire.

### Analyses

Answers were presented using numbers, percentages, means, and standard deviations. Differences between profession groups were calculated using ANOVA for continuous variables and chi-square tests for categorical variables. To analyze if MHCPs own lifestyle habits, gender, and profession were associated with taking a history on lifestyle habits and referral practices, an ordinal regression was done with the use of the following independent variables: age per 10 years, gender (female, male), BMI per 5 units, question “how important their own lifestyle is” (0–10), smoking status (smoked in past, never smoked, current smoker), alcohol use (no, yes), physical activity level (meets the physical activity criteria, meets 1 physical activity criterium, meets 0 criteria), dietary habits (has/has not a balanced eating pattern), sleep satisfaction (0–10), and profession (other, psychologist, resident in psychiatry, G-based nurse specialist, referring MHCP with nursing background, referring MHCP with a scientific background, clinical psychologist, and psychiatrist). Additionally, we did a sensitivity analysis with the variable work-related stress. Results were presented using odds ratios, confidence intervals, and significance levels. For the analyses on taking a history on tobacco and alcohol use, and advice to quit smoking, only MHCPs working with adults and elderly were included. For the analyses on referral practices, only referring MHCPs were included. A Bonferroni correction was done to correct for multiple testing, such that *p* < 0.006 was considered statistically significant. SPSS version 27.0 was used for analyses.

## Results

### Participants

Table 1 presents participant characteristics, their lifestyle habits, and their practices on taking a lifestyle history and referral to lifestyle interventions. A total of 1,607 participants filled in the online questionnaire. About 78.4% of the participants were female, age ranged between 22 and 75 years, and lifestyle interventions were available in 62.9% of the work settings. Table S1 in the Supplementary Material presents all results of the questionnaire, also divided by profession category.

**Table 1.** Participant characteristics, lifestyle habits, and lifestyle history and referral practices

Variables	*n*/mean (SD/%)
Gender, *n* (%) female	1,260 (78.4)
Age, years, mean (SD)	42.32 (11.4)
Profession, *n* (%)	
Psychologist	175 (10.9)
Resident in psychiatry	168 (10.5)
GP-based nurse specialist	211 (13.1)
Referring MHCP with nursing background	236 (14.7)
Referring MHCP with scientific background	338 (21.0)
Clinical psychologist	81 (5.0)
Psychiatrist	300 (18.7)
Other	98 (6.1)
Work setting, *n* (%)	
Psychiatric organization	1,165 (72.5)
Hospital	47 (2.9)
Academic hospital	101 (6.3)
Own practice	41 (2.6)
General practitioner’s practice	204 (12.7)
Other	48 (3.0)
Main population, *n* (%)	
Children/youth	298 (18.6)
Adults	1,048 (65.3)
Elderly	88 (5.5)
Not specifically one population	172 (10.7)
Lifestyle interventions available within work setting, *n* (%)	1,010 (62.9)
*Lifestyle habits*	
How important do you find your own lifestyle? (0–10)	8.04 (1.1)
BMI, mean (SD)	23.92 (3.6)
BMI > 24.9, *n* (%)	496 (30.9)
Current smoker, *n* (%)	87 (5.4)
Smoked in past, *n* (%)	219 (13.6)
Drinks alcohol, *n* (%)	1,161 (72.2)
Physical activity levels in minutes of moderate to vigorous physical activity per week, mean (SD)	228.80 (171.0)
Physical activity guidelines, *n* (%)	
≥150 moderate to vigorous physical activity minutes per week	1,047 (65.2)
≥2x bone and muscle strengthening activities per week	957 (59.6)
Both	736 (45.8)
Do you have a balanced eating pattern? *Yes*	1,414 (88.0)
How satisfied are you with your sleep? (0–10)	6.99 (1.6)
How much stress do you experience at work? (0–10)	5.52 (2.0)
*Lifestyle history and referring practices*	
What proportion of your patients do you ask about:	
Physical activity habits	58.16 (28.4)
Their dietary habits	55.06 (28.5)
Their sleeping pattern	83.97 (16.0)
*Only MHCPs of adult patients*	
Tobacco use	72.55 (29.8)
Alcohol use	81.78 (21.2)
*Only MHCPs of adult patients* What proportion of your patients who smoke do you advise to quit smoking?	44.16 (31.3)
What proportion of your patients do you refer to a lifestyle intervention to improve their mental health?	41.07 (27.6)
What proportion of your patients do you refer to a lifestyle intervention to improve their psychical health?	37.91 (26.7)
What proportion of your patients with sleeping problems do you refer to insomnia CBT/do you give insomnia CBT yourself?	29.70 (25.8)

MHCP, mental healthcare professional; n, number; SD, standard deviation.

### Lifestyle habits of the MHCP

On average, participants rated an 8.0 on the question if they find their own lifestyle important. BMI ranged between 15.8 and 44.4, and 30.9% of the participants were overweight. A minority (5.4%) are current smokers, and 13.6% smoked in the past. The majority of the participants (72.2%) drinks alcohol and 6.8% uses drugs. About 45.8% of the MHCPs adheres to the physical activity guidelines, 88.0% believes they have a balanced eating pattern, and the MHCPs rate their sleep on average a 7.0 out of 10. The average level of stress at work is rated a 5.5 out of 10.

### Lifestyle within psychiatric treatment

MHCPs rated on average an 8.1 to how important lifestyle is in psychiatric treatment and 87.6% (totally) agreed with the statement that lifestyle should be part of every psychiatric treatment. They found that the responsibility for the lifestyle treatment of a psychiatric patient lies with every MHCP (77.0%), followed by the patient (66.0%) and the general practitioner (43.6%).

MHCPs asked patients the most about their sleeping pattern (84.0%), followed by alcohol use (81.2%), tobacco use (72.6%), physical activity (58.2%) and dietary habits (55.1%). They advised 44.2% of their patients to quit smoking if they smoke. Moreover, they referred 41.1% of their patients to lifestyle interventions to improve their mental health, and 37.9% of their patients were referred to lifestyle interventions to improve their physical health. MHCPs referred 29.7% of their patients to insomnia CBT or gave insomnia CBT themselves if patients experience sleep problems.

### Association between personal lifestyle habits, gender, and profession and clinical lifestyle practices

**Figure 1.**
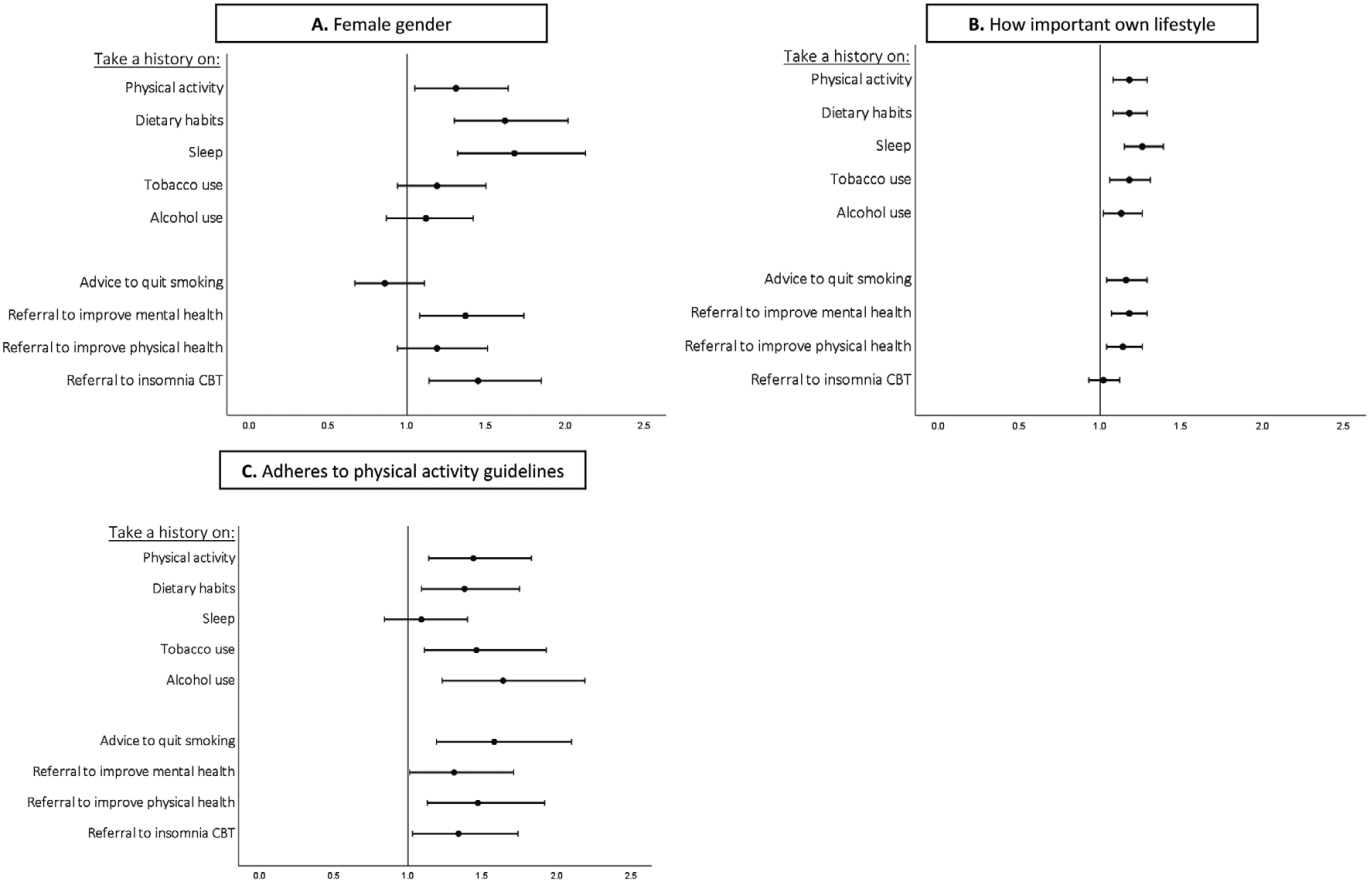
Forest plots of the results of ordinal regression analyses on MHCPs’ gender (A), importance of own lifestyle (B) and physical activity (C), and lifestyle history and referral practices. Odds ratio 0–1: take less history on lifestyle habits, advice less to quit smoking, and refer less to lifestyle interventions. Odds ratio >1: take more history on lifestyle factors, advice more to quit smoking, and refer more to lifestyle interventions.

**Figure 2.**
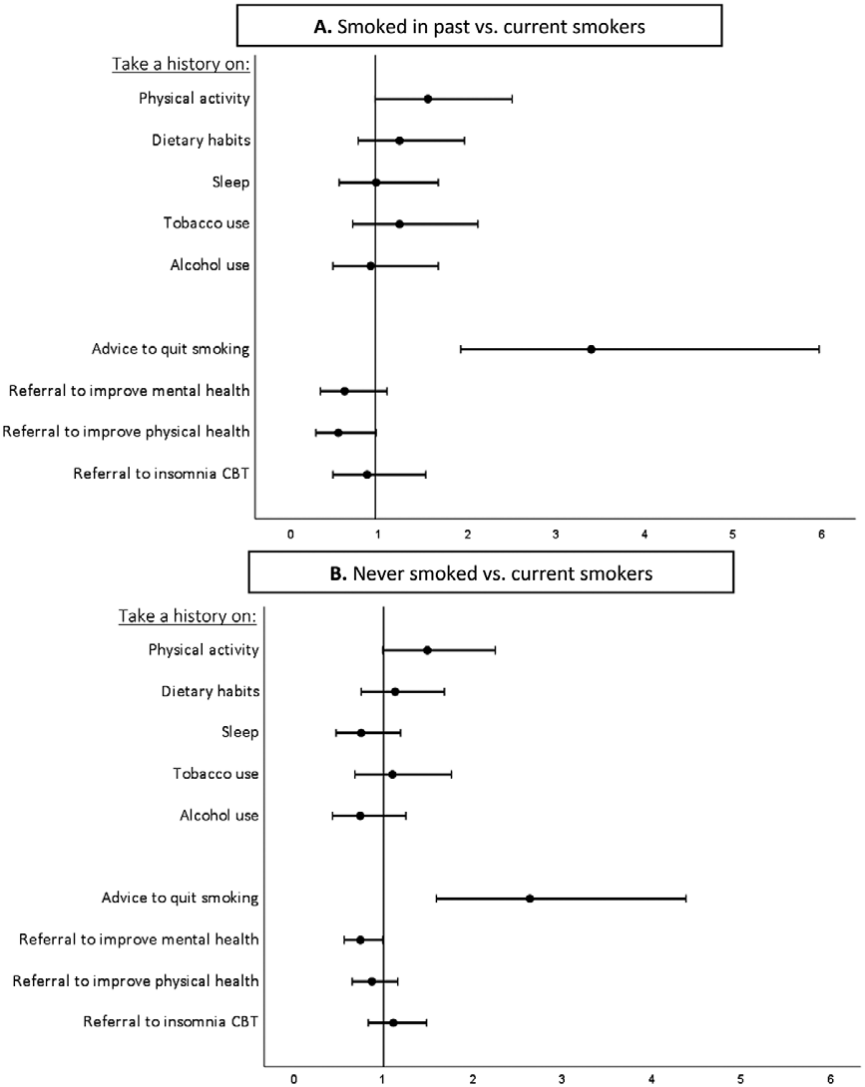
Forest plots of the results of ordinal regression analyses on MHCPs’ smoking status, and lifestyle history and referral practices. A: MHCPs who smoked in the past versus current smokers. B: MHCPs who never smoked versus current smokers. Odds ratio 0–1: take less history on lifestyle habits, advice less to quit smoking, and refer less to lifestyle interventions. Odds ratio >1: take more history on lifestyle factors, advice more to quit smoking, and refer more to lifestyle interventions.

Table 2 and Figures 1 and 2 show the results of the ordinal regression analyses on lifestyle habits, gender, profession, and lifestyle history and referral practices. MHCPs who find their own lifestyle important have higher odds to take a lifestyle history, to advice patients to quit smoking and to refer patients to lifestyle interventions. Compared to current smokers, MHCPs who smoked in the past have higher odds to take a history on physical activity, and MHCPs who smoked in the past and MHCPs who never smoked have higher odds to advice patients to quit smoking. Physically active MHCPs have higher odds to take a history on lifestyle habits, except for sleep, and higher odds to advice patients to quit smoking, and to refer to lifestyle interventions. MHCPs who have a balanced eating pattern have higher odds to ask patients about physical activity, and MHCPs who are more satisfied with their sleep have higher odds to ask patients about their sleep pattern, and to refer patients to lifestyle interventions to improve their mental health. Female MHCPs have higher odds to take a history on physical activity, dietary habits and sleep, and higher odds to refer patients to lifestyle interventions to improve their mental health and sleep.

**Table 2.** Results of the ordinal regression analyses on lifestyle habits, gender, profession, and lifestyle history and referral practices

Variable	History on									
	Physical activity, *n* = 1,580		Dietary habits, *n* = 1,579		Sleep, *n* = 1,576		Tobacco use, *n* = 1,275		Alcohol, *n* = 1,208	
	OR	CI	OR	CI	OR	CI	OR	CI	OR	CI
Age per 10 years	1.13*	1.03–1.24	0.99	0.90–1.09	0.92	0.83–1.01	1.07	0.96–1.20	1.07	0.95–1.21
Female	1.31	1.05–1.64	1.62**	1.30–2.02	1.68**	1.32–2.13	1.19	0.94–1.50	1.12	0.87–1.42
BMI per 5 units	0.99	0.86–1.13	1.02	0.89–1.16	1.14	0.99–1.32	1.09	0.93–1.28	1.08	0.92–1.27
How important is own lifestyle	1.18**	1.08–1.29	1.18**	1.08–1.29	1.26**	1.15–1.39	1.18**	1.06–1.31	1.13	1.02–1.26
Smoking (reference = smoker)										
*Smoked in past*	1.59	1.00–2.52	1.27	0.81–1.99	1.01	0.60–1.70	1.27	0.75–2.14	0.95	0.53–1.70
*Never smoked*	1.49	0.99–2.25	1.13	0.75–1.68	0.75	0.47–1.19	1.10	0.68–1.76	0.74	0.43–1.25
Does not drink alcohol	0.86	0.71–1.05	0.91	0.74–1.11	1.01	0.81–1.25	1.07	0.85–1.36	1.07	0.84–1.37
Physical activity norm (reference = meets non criteria)										
*Meets 2 criteria*	1.44**	1.14–1.83	1.38*	1.09–1.75	1.09	0.84–1.40	1.46*	1.11–1.93	1.64**	1.23–2.19
*Meets 1 criterium*	1.41**	1.10–1.80	1.26	0.99–1.61	1.00	0.77–1.30	1.41	1.06–1.87	1.41	1.05–1.89
Has a balanced eating pattern	1.37	1.04–1.81	1.18	0.90–1.56	0.91	0.67–1.23	0.95	0.69–1.32	1.11	0.79–1.55
Sleep satisfaction	1.03	0.97–1.09	1.00	0.95–1.06	1.09**	1.03–1.16	1.06	0.99–1.13	1.06	1.00–1.14
Profession (reference = psychiatrist)										
*Other*	1.015	0.66–1.56	1.07	0.69–1.63	0.22**	0.13–0.35	0.51	0.30–0.87	0.22**	0.13–0.39
*Psychologist*	1.05	0.72–1.53	1.16	0.80–1.69	0.44**	0.30–0.66	0.61	0.39–0.95	0.73	0.46–1.16
*Resident in psychiatry*	0.46**	0.33–0.66	0.52**	0.36–0.74	0.72	0.49–1.06	1.03	0.69–1.54	0.70	0.46–1.07
*GP-based nurse specialist*	3.64**	2.65–5.01	1.55**	1.13–2.11	0.69	0.49–0.97	0.21**	0.15–0.30	0.21**	0.15–0.30
*Referring MHCP with nursing background*	2.90**	2.12–3.95	2.95**	2.17–4.03	1.18	0.83–1.68	1.33	0.92–1.92	1.38	0.92–2.07
*Referring MHCP with scientific background*	1.25	0.93–1.67	1.47*	1.10–1.96	0.56**	0.41–0.77	0.61*	0.43–0.87	0.75	0.51–1.09
*Clinical psychologist*	0.92	0.60–1.42	1.51	0.97–2.34	0.43**	0.28–0.68	0.45**	0.28–0.73	0.63	0.37–1.06

	Advice to quit smoking, *n* = 1,090		Referral to improve mental health, *n* = 1,305		Referral to improve physical health, *n* = 1,295		Referral to insomnia CBT, *n* = 1,294	
Variable	OR	CI	OR	CI	OR	CI	OR	CI
Age per 10 years	0.96	0.86–1.08	1.07	0.97–1.19	1.05	0.95–1.17	1.05	0.94–1.16
Female	0.86	0.67–1.11	1.37*	1.08–1.74	1.19	0.94–1.51	1.45**	1.14–1.85
BMI per 5 units	0.95	0.80–1.12	0.91	0.78–1.05	0.91	0.78–1.05	0.92	0.79–1.06
How important is own lifestyle	1.16*	1.04–1.29	1.18**	1.07–1.29	1.14*	1.04–1.26	1.02	0.93–1.12
Smoking (reference = smoker)								
*Smoked in past*	3.40**	1.95–5.93	0.66	0.39–1.13	0.59	0.34–1.01	0.91	0.53–1.56
*Never smoked*	2.64**	1.59–4.38	0.74	0.56–0.99	0.87	0.65–1.16	1.11	0.83–1.48
Does not drink alcohol	1.21	0.95–1.55	0.83	0.67–1.04	0.83	0.67–1.04	1.06	0.85–1.32
Physical activity norm (reference = meets non criteria)								
*Meets 2 criteria*	1.58**	1.19–2.10	1.31	1.01–1.71	1.47**	1.13–1.92	1.34	1.03–1.74
*Meets 1 criterium*	1.58**	1.18–2.12	1.37	1.05–1.80	1.49**	1.13–1.95	1.19	0.91–1.55
Has a balanced eating pattern	0.85	0.61–1.17	0.98	0.72–1.33	0.93	0.69–1.27	1.08	0.79–1.47
Sleep satisfaction	1.01	0.95–1.08	1.07	1.01–1.14	1.02	0.96–1.08	1.02	0.96–1.09
Profession (reference = psychiatrist)								
*Resident in psychiatry*	0.71	0.48–1.04	0.72	0.50–1.05	0.67	0.46–0.97	0.78	0.53–1.14
*GP-based nurse specialist*	0.47**	0.33–0.66	1.35	0.98–1.86	1.27	0.92–1.76	1.73**	1.25–2.39
*Referring MHCP with nursing background*	1.11	0.79–1.54	1.50*	1.09–2.05	1.78**	1.30–2.44	1.08	0.78–1.48
*Referring MHCP with scientific background*	0.31**	0.22–0.43	0.67*	0.50–0.90	0.66*	0.49–0.89	0.81	0.60–1.08
*Clinical psychologist*	0.31**	0.19–0.52	0.59	0.38–0.92	0.78	0.50–1.22	1.32	0.87–2.02

CI, confidence interval; GP, general practitioner; MHCP, mental healthcare professional; n, number; OR, odds ratio.

* *p* < 0.01.

** *p* < 0.006.

Psychologists, residents in psychiatry, referring MHCPs with a scientific background, and clinical psychologists had lower odds to take a history on several lifestyle habits, and referring MHCPs with a scientific background and clinical psychologists had lower odds to refer patients to lifestyle interventions. On the contrary, MHCPs with a nursing background had higher odds to take a history on several lifestyle habits, and higher odds to refer patients to lifestyle interventions compared to psychiatrists. Interestingly, GP-based nurse practitioners had lower odds to take a history on sleep, tobacco, and alcohol use, but higher odds to take a history on physical activity and dietary habits than psychiatrists. In addition, they had lower odds to advice patients to quit smoking, but higher odds to refer patients to insomnia CBT.

The sensitivity analysis on work related stress did not change these outcomes (Table S2 in the Supplementary Material).

## Discussion

We found that Dutch MHCPs are often non-smokers (94.6%), but the majority does consume alcohol (72.2%). Less than half (45.8%) adheres to the physical activity guidelines, 88.0% believes they have a balanced eating pattern, and they rate their sleep on average a 7.0 out of 10. The majority (87.6%) finds that lifestyle should be part of every psychiatric treatment, but depending on which lifestyle factor, 55.1–84.0% take a lifestyle history, 29.7–41.1% refer to interventions, and less than half (44.2%) of smoking patients are advised to quit. MHCPs who find their lifestyle important, who are physically more active, females, and MHCPs with a nursing background take more lifestyle histories and refer more often. Compared to current smokers, MHCPs who never or formerly smoked have higher odds (2.64 and 3.40, respectively, *p* < 0.001) to advice patients to quit smoking.

The physical activity and alcohol consumption are representative for the Dutch population [24]. Yet, our sample smokes less (5.4% vs. 26.1%), is less overweight (31.6% vs. 51.6%), and eats more fruit on a daily basis (56.4% vs. 26.0%) [24], which might also be explained by the younger age of our population. In the context of similar evaluations, the sample is more active than Australian psychiatrists [11], corresponding to previous research [20].

Noteworthy, less than half of the patients who smoke are advised to quit, while the Very Brief Advice, an evidence-based intervention to increase quit smoking attempts, only costs 30 s of time [25]. Surprisingly, MHCPs refer the least to insomnia CBT or give insomnia CBT themselves, while sleep problems occur often in patients with mental illness, and insomnia CBT is an effective treatment that also decreases psychiatric symptoms [6, 26].

In addition, we found that MHCPs who find their own lifestyle important, who are physically more active and female MHCPs more often take lifestyle histories and refer more frequently to lifestyle interventions. Compared to current smokers, MHCPs who formerly or never smoked have higher odds to advice patients to quit smoking. These findings are in line with previous research in psychiatry [20] and general healthcare [12–15, 17–19]. Interestingly, MHCPs tend to take more histories and make more referrals if they engage in the lifestyle behavior themselves. This could be explained by the fact that it is easier to talk about and advise patients on behavior that you personally consider important and engage in yourself. Regarding profession groups, it was noticeable that professionals with less clinical experience and medical training did take less lifestyle history and referred less, and MHCPs with a nursing background did more in comparison to psychiatrists.

Our findings show that taking a lifestyle history and referral depends on the lifestyle habits, gender, and profession of the MHCP. There is compelling evidence that lifestyle interventions can improve mental and physical health and these interventions are therefore recommended in the treatment for people with mental illness [5–10]. Therefore, taking a lifestyle history should always be done, and referral to lifestyle interventions should be considered in every psychiatric treatment, and should not depend on the personal and professional characteristics of the MHCP. Previous research on counseling of physicians in primary care suggested that physicians often struggle with counseling on health behavior that they do not practice themselves [27]. It seems therefore important to practice what you preach, also since patients see their physicians as role models for healthy lifestyle habits, and physicians who disclose information about their own lifestyle are found to be more credible and motivating [28].

To improve the clinical lifestyle practices of MHCPs at a short notice, it is important to educate MHCPs on the evidence about lifestyle psychiatry, lifestyle counseling and referral. Practical training in motivational interviewing can be particularly helpful in this regard. In addition, practical guidelines and referral options should be provided. Currently, there are hardly any courses on healthy lifestyle in education and medical training [29], and lifestyle interventions are often not part of treatment guidelines. Our finding that MHCPs with less clinical experience and medical training take less lifestyle histories and refer less, underline the necessity of adequate training. Next to this, it might not only be important to look at the MHCP, but also to critically review the role of healthy lifestyle in the healthcare system and society. In most healthcare systems, just as in The Netherlands, financial incentives in healthcare are based on the treatment of diseases, and not on the prevention of diseases [30]. Moreover, medical insurance only reimburse a few lifestyle interventions, and a mental illness is not an indication for referral to these lifestyle interventions [31]. Future research should investigate barriers and facilitators MHCPs experience in taking lifestyle histories and referring to lifestyle interventions to identify solutions to improve their clinical lifestyle practices.

### Limitations

Our study has some limitations. Our main concern is that there might have been selection bias, since MHCPs who believe lifestyle is an important topic within psychiatry, and MHCPs who have a healthier lifestyle might be more likely to fill in the questionnaire. To reduce this bias, we especially stated in the invitation, that all MHCPs, also when they believe healthy lifestyle should not play a role in psychiatry, were invited to participate. That healthier and more lifestyle-focused MHCPs might have participated in this study may also indicate that the actual clinical lifestyle practices are even worse. Moreover, this limitation is inherent to questionnaire-focused research and it will be challenging to study this topic in a more objective way. After all, this is the largest sample including these data and thereby the most representative, so far. Nevertheless, this remains a cross-sectional study, thus we only examined associations and not causal relations. Another limitation is that the questionnaire was a self-reporting questionnaire, and we did not measure lifestyle habits by performing for example physical activity tracking. Therefore, the actual lifestyle habits of the MHCP might differ. Lastly, we framed the question on referral to lifestyle interventions as “if you believe there is something to gain for the patient in terms of lifestyle?” This could have caused bias because the response depends on what the participant perceives as a healthy lifestyle.

This study indicates that MHCPs’ personal lifestyle habits, gender, and profession affect their clinical lifestyle practices, and thereby the translation of compelling evidence on lifestyle psychiatry to improved healthcare for patients.

## Supporting information

Koomen et al. supplementary material Koomen et al. supplementary material

Koomen et al. supplementary material Koomen et al. supplementary material

## Supplementary material

The supplementary material for this article can be found at https://doi.org/10.1192/j.eurpsy.2023.2475.

## References

[1] Patel V, Saxena S, Lund C, Thornicroft G, Baingana F, Bolton P, et al. The Lancet Commission on global mental health and sustainable development. Lancet. 2018;392:1553–98. 30314863 10.1016/S0140-6736(18)31612-X

[2] GBD 2019 Mental Disorders Collaborators. Global, regional, and national burden of 12 mental disorders in 204 countries and territories, 1990–2019: a systematic analysis for the global burden of disease study 2019. Lancet Psychiatry. 2022;9(2):137–50. 35026139 10.1016/S2215-0366(21)00395-3 PMC8776563

[3] Plana-Ripoll O, Weye N, Momen NC, Christensen MK, Iburg KM, Laursen TM, et al. Changes over time in the differential mortality gap in individuals with mental disorders. JAMA Psychiatry. 2020;77(6):648–50. 32267492 10.1001/jamapsychiatry.2020.0334 PMC7142807

[4] Plana-Ripoll O, Pedersen CB, Agerbo E, Holtz Y, Erlangsen A, Canudas-Romo V, et al. A comprehensive analysis of mortality-related health metrics associated with mental disorders: a nationwide, register-based cohort study. Lancet. 2019;394(10211):1827–35. 31668728 10.1016/S0140-6736(19)32316-5

[5] Firth J, Siddiqi N, Koyanagi A, Siskind D, Rosenbaum S, Galletly C, et al. The lancet psychiatry commission: a blueprint for protecting physical health in people with mental illness. Lancet Psychiatry. 2019;6:675–712. 31324560 10.1016/S2215-0366(19)30132-4

[6] Firth J, Solmi M, Wootton R, Vancampfort D, Schuch F, Gilbody S, et al. A meta-review of “lifestyle psychiatry”: the role of exercise, smoking, diet and sleep in the prevention and treatment of mental disorders. World Psychiatry. 2020;19:360–80. 32931092 10.1002/wps.20773 PMC7491615

[7] Stubbs B, Vancampfort D, Hallgren M, Firth J, Veronese N, Solmi M, et al. EPA guidance on physical activity as a treatment for severe mental illness: a meta-review of the evidence and position statement from the European psychiatric association (EPA), supported by the International Organization of Physical Therapists in mental health (IOPTMH). Eur Psychiatry. 2018;54:124–44. 30257806 10.1016/j.eurpsy.2018.07.004

[8] World Health Organization. Management of physical health conditions in adults with severe mental disorders WHO GUIDELINES. Geneva: World Health Organization; 2018. Available from: https://iris.who.int/bitstream/handle/10665/275718/9789241550383-eng.pdf?sequence=1 30507109

[9] NICE. Physical activity: brief advice for adults in primary care [Internet]. 2013. Available from: www.nice.org.uk/guidance/ph44.

[10] NICE. Psychosis and schizophrenia in adults quality standard [Internet]. 2015. Available from: www.nice.org.uk/guidance/qs80.

[11] Fibbins H, Czosnek L, Stanton R, Davison K, Lederman O, Morell R, et al. Self-reported physical activity levels of the 2017 Royal Australian and New Zealand College of Psychiatrists (RANZCP) conference delegates and their exercise referral practices. J Ment Health. 2020;29(5):565–72. 30322334 10.1080/09638237.2018.1521935

[12] Carlos S, Carlos S, Rico-Campà A, Rico-Campà A, Rico-Campà A, De La Fuente-Arrillaga C, et al. Do healthy doctors deliver better messages of health promotion to their patients?: Data from the SUN cohort study. Eur J Pub Health. 2020;30(3):466–72. 32060517 10.1093/eurpub/ckaa019

[13] Abramson S, Stein J, Schaufele M, Frates E, Rogan S. Personal exercise habits and counseling practices of primary care physicians: a national survey. Clin J Sport Med. 2000;10:40–8. 10.1097/00042752-200001000-00008 10695849

[14] Brotonsc C, Björkelund C, Bulc M, Ciurana R, Godycki-Cwirko M, Jurgova E, et al. Prevention and health promotion in clinical practice: the views of general practitioners in Europe. Prev Med (Baltim). 2005;40(5):595–601. 10.1016/j.ypmed.2004.07.020 15749144

[15] Belfrage ASV, Grotmol KS, Tyssen R, Moum T, Finset A, Rø KI, et al. Factors influencing doctors’ counselling on patients’ lifestyle habits: a cohort study. BJGP Open. 2018;2(3):bjgpopen18X101607. 10.3399/bjgpopen18X101607 PMC6202006 30564740

[16] Zhu DQ, Norman IJ, While AE. The relationship between doctors’ and nurses’ own weight status and their weight management practices: a systematic review. Obes Rev. 2011;12(6):459–69. 21366835 10.1111/j.1467-789X.2010.00821.x

[17] Fie S, Norman IJ, While AE. The relationship between physicians’ and nurses’ personal physical activity habits and their health-promotion practice: a systematic review. Health Educ J. 2013;72:102–19.

[18] Frank E, Segura C, Shen H, Oberg E. Predictors of Canadian physicians’ prevention counseling practices. Can J Public Health. 2010;101(5):390–5. 21214054 10.1007/BF03404859 PMC6974278

[19] Lewis CE, Clancy C, Leake B, Schwartz JS. The counseling practices of internists. Ann Intern Med. 1991;114:54–8. 1983933 10.7326/0003-4819-114-1-54

[20] Deenik J, Koomen LEM, Scheewe TW, van Deursen FP, Cahn W. Cardiorespiratory fitness and self-reported physical activity levels of referring mental healthcare professionals, and their attitudes and referral practices related to exercise and physical health. J Psychiatr Res. 2022;154:19–27. 35921725 10.1016/j.jpsychires.2022.07.029

[21] Wilson DMC, Nielsen E, Ciliska D. Lifestyle assessment: testing the FANTASTIC instrument. Can Fam Physician. 1984;30:1863–6.

[22] Bull FC, Al-Ansari SS, Biddle S, Borodulin K, Buman MP, Cardon G, et al. World Health Organization 2020 guidelines on physical activity and sedentary behaviour. Br J Sports Med. 2020;54(24):1451–62. 33239350 10.1136/bjsports-2020-102955 PMC7719906

[23] WHO. WHO STEPS surveillance manual. 2020. Available from: https://cdn.who.int/media/docs/default-source/ncds/ncd-surveillance/steps/steps-manual.pdf?sfvrsn=c281673d_8

[24] Kraaykamp G, André S, Meuleman R. Gezondheidsgedrag in Nederland. 2018. Available from: https://digitaal.scp.nl/leefstijl/gezondheidsgedrag-in-nederland/.

[25] Aveyard P, Begh R, Parsons A, West R. Brief opportunistic smoking cessation interventions: a systematic review and meta-analysis to compare advice to quit and offer of assistance. Addiction. 2012;107:1066–73. 22175545 10.1111/j.1360-0443.2011.03770.x

[26] Freeman D, Sheaves B, Waite F, Harvey AG, Harrison PJ. Sleep disturbance and psychiatric disorders. Lancet Psychiatry. 2020;7(7):628–37. 32563308 10.1016/S2215-0366(20)30136-X

[27] Vickers KS, Kircher KJ, Smith MD, Petersen LR, Rasmussen NH. Health behavior counseling in primary care: provider-reported rate and confidence. Fam Med. 2007;39:730–5. 17987416

[28] Frank E, Breyan J, Elon L. Physician disclosure of healthy personal behaviors improves credibility and ability to motivate. Arch Fam Med. 2000;9:287–90. 10728118 10.1001/archfami.9.3.287

[29] Stichting Student en Leefstijl. Voeding en leefstijl in het Geneeskunde curriculum. 2019. Available from: https://www.studentenleefstijl.nl/wp-content/uploads/2020/11/Kennissynthese.pdf

[30] Varkevisser M, Schut E, Franken F, Van Der Geest S. Sustainability and resilience in the Dutch health system: The Netherlands [Internet]. 2023. Available from: www.phssr.org.

[31] Rijksinstituut voor Volksgezondheid en Milieu. Gecombineerde leefstijlinterventie. 2023. Available from: https://www.loketgezondleven.nl/gezondheidsthema/overgewicht/gecombineerde-leefstijlinterventie.

