# Supplementary material for: The association between mental healthcare professionals’ personal characteristics and their clinical lifestyle practices: a national cross-sectional study in The Netherlands
Source: Eur Psychiatry. 2023 Dec 4;66(1):e96. doi: 10.1192/j.eurpsy.2023.2475 (PMC10755571; doi:10.1192/j.eurpsy.2023.2475)
Supplement: Koomen et al. supplementary material [file S0924933823024756sup001.docx]

**Supplementary material**

**Questionnaire**

1. What is your age?
2. What is your sex?
   1. Male. B. Female. C. Other
3. What is your profession?
   1. General practice-based nurse specialist. B. Specialized nurse. C. Psychologist. D. Healthcare psychologist. E. Clinical psychologist. F. Psychiatry resident. G. Psychiatrist. E.Other
      1. If other, what is your profession? (open question)
4. Where do you mainly work?
   1. Organization for psychiatric care. B. Hospital. C. Academic hospital. D. Own practice. E. General practitioners practice. F. Other
      1. If other, where do you work? (open question)
5. What is your main population?
   1. Children/youth. B. Adults. C. Elderly. D. Not specifically one population
6. Are lifestyle interventions available within your work setting?
   1. If yes: what kind of lifestyle interventions?
7. What is your height?
8. What is your weight?
9. Do you smoke?
   1. Yes
      1. How many do you smoke a day?
         1. 1. B. 1-5. C. 5-10. D. 10-20. E. >20
   2. No
   3. I have smoked in the past
      1. How many years ago did you quit smoking?
10. Do you drink alcohol?
    1. Yes
       1. How many alcohol units do you drink per week?
          1. <1. B. 1. C. 2-3. D. 3-5. E. 5-7. F. 7-10. G. 10-15. H. 15-20. I. >20
    2. No
11. Did you have an alcohol problem in the past?
12. Do you use drugs?
    1. Yes
       1. How often do you use drugs?
          1. Daily. B. Weekly. C. Monthly. D. Several time a year. E. Yearly. F. Less than yearly
    2. No
13. Did you have a problem with drugs in the past?
14. How important do you find your own lifestyle on a scale of 0-10?
15. How many days per week on average do you do physical activity of medium or high intensity (with increased heart rate/respiration, e.g. brisk walking, cycling, team sports)?
16. What kind of physical activities do you do?
    1. Walking. B. Bicycling. C. Running. D. Cycling. E. Gym. F. Team sports. G. Tennis. H. Golf. I. Boxing. J. Swimming. K. Crossfit. L. Other
       1. What? (open question)
17. How many minutes per week do you spend on these activities? (open question)
18. How many days per week on average do you do muscle and bone strengthening exercises? (0-7)
19. How fit do you consider yourself on a scale of 1-10?
20. Do you have a balanced eating pattern?
    1. Yes. B. No
21. Do you eat an average of 2 pieces of fruit per day?
    1. Yes. B. No. C. Sometimes
22. How many days a week do you eat vegetables? (0-7)
23. Have you been treated by a dietitian?
    1. Yes. B. No
24. How satisfied are you with your sleep?
    1. Scale 0-10 (0 = very unsatisfied, 10 = very satisfied)
25. How much stress do you experience at work?
    1. Scale 0-10 (0 = no stress, 10 = a lot of stress)
26. How much stress do you experience at home?
    1. Scale 0-10 (0 = no stress, 10 = a lot of stress)
27. How important do you find lifestyle in psychiatric treatment?
    1. Scale 0-10 (0 = not important at all, 10 = very important)
28. Statement: lifestyle should be part of any psychiatric treatment
    1. Totally disagree. B. Disagree. C. Neutral. D. Agree. E. Totally agree
29. Who do you think is responsible for the lifestyle treatment of the psychiatric patient?
    1. General practitioner. B. Main responsible psychiatric practitioner. C. Every psychiatric practitioner. D. Personal care givers or nurses. E. Patient. F. Other
       1. Who? (open question)
30. What proportion of your patients do you ask about smoking habits?
    1. O%. B. 0-10%. C. 10-20%. D. 20-30%. E. 30-40%. F. 40-50%. G. 50-60%. H. 60-70%. I. 70-80%. J. 80-90%. K. 90-100%. L. Not applicable
31. What proportion of your patients do you ask about alcohol use?
    1. O%. B. 0-10%. C. 10-20%. D. 20-30%. E. 30-40%. F. 40-50%. G. 50-60%. H. 60-70%. I. 70-80%. J. 80-90%. K. 90-100%. L. Not applicable
32. What proportion of your patients do you ask about physical activity habits?
    1. O%. B. 0-10%. C. 10-20%. D. 20-30%. E. 30-40%. F. 40-50%. G. 50-60%. H. 60-70%. I. 70-80%. J. 80-90%. K. 90-100%. L. Not applicable
33. What proportion of your patients do you ask about their dietary habits?
    1. O%. B. 0-10%. C. 10-20%. D. 20-30%. E. 30-40%. F. 40-50%. G. 50-60%. H. 60-70%. I. 70-80%. J. 80-90%. K. 90-100%. L. Not applicable
    2. What O%. B. 0-10%. C. 10-20%. D. 20-30%. E. 30-40%. F. 40-50%. G. 50-60%. H. 60-70%. I. 70-80%. J. 80-90%. K. 90-100%. L. Not applicable
34. What proportion of your patients who smoke do you advise to stop smoking?
    1. O%. B. 0-10%. C. 10-20%. D. 20-30%. E. 30-40%. F. 40-50%. G. 50-60%. H. 60-70%. I. 70-80%. J. 80-90%. K. 90-100%. L. Not applicable
35. What proportion of your patients do you refer to ‘an intervention aimed at improving or maintaining someone's lifestyle’ to improve their MENTAL health, if you believe there is something to gain for the patient in terms of lifestyle?
    1. O%. B. 0-10%. C. 10-20%. D. 20-30%. E. 30-40%. F. 40-50%. G. 50-60%. H. 60-70%. I. 70-80%. J. 80-90%. K. 90-100%. L. Not applicable
36. Where do you refer to?
    1. Psychomotor therapist. B. Physiotherapy. C. Physical activity worker. D. Regular sports activities. E. Dietician. F. Other
       1. Where? (open question)
37. What proportion of your patients do you refer to ‘an intervention aimed at improving or maintaining someone's lifestyle’ to improve their PHYSICAL health, if you believe there is something to gain for the patient in terms of lifestyle?
38. Where do you refer to?
    1. Psychomotor therapist. B. Physiotherapy. C. Physical activity worker. D. Regular sports activities. E. Dietician. F. Other
       1. Where? (open question)
39. What proportion of your patients with sleeping problems do you refer to insomnia CBT/do you give insomnia CBT yourself?
    1. O%. B. 0-10%. C. 10-20%. D. 20-30%. E. 30-40%. F. 40-50%. G. 50-60%. H. 60-70%. I. 70-80%. J. 80-90%. K. 90-100%. L. Not applicable
40. Do you want to mention anything else?

|  | | | | | | | | | |  |
| --- | --- | --- | --- | --- | --- | --- | --- | --- | --- | --- |
| ***Variables*** | **Total (N=1607)** | **Psychologist (N=175)** | **Resident In psychiatry (N=168)** | **GP based nurse specialist (N=211)** | **Referring MHCP with nursing background (N=236)** | **Referring MHCP with scientific background (N=338)** | **Clinical psychologist (N=81)** | **Psychiatrist (N=300)** | **Other (N=98)** | **p-value** |
| Sex, n (%) female | 1260 (78.4) | 161 (92.0) | 111 (66.1) | 181 (85.8) | 178 (75.4) | 298 (88.2) | 62 (76.5) | 180 (60.0) | 89 (90.8) | <0.001 |
| Age, years, mean (SD) | 42.3 (11.4) | 32.0 (7.5) | 31.9 (5.8) | 48.8 (10.1) | 46.3 (10.1) | 39.4 (9.0) | 50.0 (9.2) | 48.3 (11.2) | 42.2 (10.5) | <0.001 |
| Work setting, n (%) |  |  |  |  |  |  |  |  |  | <0.001 |
| Psychiatric organization | 1165 (72.5) | 151 (86.3) | 117 (69.6) | 6 (2.8) | 209 (88.6) | 307 (90.8) | 68 (84.0) | 222 (74.2) | 85 (86.7) |  |
| Hospital | 47 (2.9) | 3 (1.7) | 10 (6.0) | 0 (0.0) | 4 (1.7) | 9 (2.7) | 2 (2.5) | 18 (6.0) | 1 (1.0) |  |
| Academic hospital | 101 (6.3) | 7 (4.0) | 40 (23.8) | 0 (0.0) | 12 (5.1) | 5 (1.5) | 3 (3.7) | 28 (9.4) | 6 (6.1) |  |
| Own practice | 41 (2.6) | 4 (2.3) | 0 (0.0) | 2 (0.9) | 2 (0.8) | 12 (3.6) | 4 (4.9) | 17 (5.7) | 0 (0.0) |  |
| General practitioner’s practice | 204 (12.7) | 1 (0.6) | 0 (0.0) | 199 (94.3) | 1 (0.4) | 1 (0.3) | 0 (0.0) | 0 (0.0) | 2 (2.0) |  |
| Other | 48 (3.0) | 9 (5.1) | 1 (0.6) | 4 (1.9) | 8 (3.4) | 4 (1.2) | 4 (4.9) | 14 (4.7) | 4 (4.1) |  |
| Main population, n (%) |  |  |  |  |  |  |  |  |  | <0.001 |
| Children/youth | 298 (18.6) | 50 (28.6) | 12 (7.1) | 9 (4.3) | 23 (9.7) | 122 (36.1) | 16 (19.8) | 40 (13.4) | 26 (26.5) |  |
| Adults | 1048 (65.3) | 121 (69.1) | 123 (73.2) | 131 (62.1) | 164 (69.5) | 195 (57.7) | 56 (69.1) | 204 (68.2) | 54 (55.1) |  |
| Elderly | 88 (5.5) | 2 (1.1) | 12 (7.1) | 0 (0.0) | 26 (11.0) | 12 (3.6) | 3 (3.7) | 26 (8.7) | 7 (7.1) |  |
| Not specifically one population | 172 (10.7) | 2 (1.1) | 21 (12.5) | 71 (33.6) | 23 (9.7) | 9 (2.7) | 6 (7.4) | 29 (9.7) | 11 (11.2) |  |
| Lifestyle interventions available within work setting, n (%) | 1010 (62.9) | 93 (53.1) | 116 (69.0) | 98 (46.4) | 172 (72.9) | 191 (56.5) | 57 (70.4) | 213 (71.2) | 70 (71.4) | <0.001 |
| **Own lifestyle** | | | | | | | | | | |
| BMI, mean (SD) | 23.9 (3.6) | 22.9 (3.4) | 22.9 (3.4) | 24.7 (3.8) | 25.2 (3.8) | 23.4 (3.4) | 23.9 (3.0) | 23.9 (3.5) | 24.5 (3.7) | <0.001 |
| BMI >24.9 | 496 (30.9) | 35 (20) | 36 (21.4) | 89 (42.2) | 105 (44.5) | 81 (24.0) | 28 (34.6) | 87 (29.2) | 35 (35.7) | <0.001 |
| Current smoker, n (%) | 87 (5.4) | 10 (5.7) | 8 (4.8) | 7 (3.3) | 21 (8.9) | 18 (5.3) | 2 (2.5) | 8 (2.7) | 13 (13.3) | <0.001 |
| Smoked in past, n (%) | 219 (13.6) | 12 (6.9) | 12 (7.1) | 33 (15.6) | 59 (25.0) | 31 (9.2) | 12 (14.8) | 41 (13.7) | 19 (19.4) | <0.001 |
| Drinks alcohol, n (%) | 1161 (72.2) | 128 (73.1) | 128 (76.2) | 137 (64.9) | 168 (71.2) | 247 (73.1) | 58 (71.6) | 223 (74.3) | 72 (73.5) | 0.33 |
| Uses drugs, n (%) | 109 (6.8) | 19 (10.9) | 31 (18.5) | 5 (2.4) | 14 (5.9) | 18 (5.3) | 1 (1.2) | 12 (4.0) | 9 (9.2) | <0.001 |
| PA levels, minutes MVPA per week, median | 228.8 (171.0) | 233.8 (146.2) | 222.1 (161.8) | 225.5 (168.8) | 241.2 (156.8) | 220.7 (162.4) | 236.8 (194.4) | 223.0 (201.5) | 247.3 (173.5) | 0.76 |
| Kind of activities, n (%) |  |  |  |  |  |  |  |  |  |  |
| Walking | 1073 (66.8) | 109 (62.3) | 57 (57.7) | 162 (76.8) | 165 (69.9) | 217 (64.2) | 55 (67.9) | 189 (63.0) | 79 (80.6) | <0.001 |
| Bicycling | 1023 (63.7) | 101 (57.7) | 117 (69.6) | 126 (59.7) | 146 (61.9) | 227 (67.2) | 55 (67.9) | 189 (63.0) | 62 (63.3) | 0.22 |
| Running | 490 (30.5) | 54 (30.9) | 72 (42.9) | 45 (21.3) | 69 (29.2) | 107 (31.7) | 30 (37.0) | 86 (28.7) | 27 (27.6) | <0.001 |
| Cycling | 165 (10.3) | 19 (10.9) | 28 (16.7) | 13 (6.2) | 24 (10.2) | 33 (9.8) | 10 (12.3) | 28 (9.3) | 10 (10.2) | 0.10 |
| Gym | 455 (28.3) | 74 (42.3) | 50 (29.8) | 54 (25.6) | 68 (28.8) | 87 (25.7) | 14 (17.3) | 77 (25.7) | 31 (31.6) | <0.001 |
| Team sports | 99 (6.2) | 19 (10.9) | 7 (4.2) | 8 (3.8) | 14 (5.9) | 27 (8.0) | 4 (4.9) | 12 (4.0) | 8 (8.2) | 0.03 |
| Tennis | 126 (7.8) | 21 (12.0) | 14 (8.3) | 21 (10.0) | 9 (3.8) | 30 (8.9) | 3 (3.7) | 21 (7.0) | 7 (7.1) | 0.06 |
| Golf | 15 (0.9) | 0 (0.0) | 1 (0.6) | 3 (1.4) | 1 (0.4) | 1 (0.3) | 0 (0.0) | 9 (3.0) | 0 (0.0) | 0.01 |
| Boxing | 55 (3.4) | 9 (5.1) | 6 (3.6) | 4 (1.9) | 13 (5.5) | 8 (2.4) | 3 (3.7) | 8 (2.7) | 4 (4.1) | 0.33 |
| Swimming | 163 (10.1) | 20 (11.4) | 13 (7.7) | 25 (11.8) | 22 (9.3) | 33 (9.8) | 12 (14.8) | 31 (10.3) | 7 (7.1) | 0.64 |
| CrossFit | 56 (3.5) | 10 (5.7) | 5 (3.0) | 1 (0.5) | 11 (4.7) | 17 (5.0) | 2 (2.5) | 8 (2.7) | 2 (2.0) | 0.07 |
| Bootcamp | 115 (7.2) | 21 (12.0) | 13 (7.7) | 14 (6.6) | 8 (3.4) | 34 (10.1) | 4 (4.9) | 16 (5.3) | 5 (5.1) | 0.01 |
| Other | 378 (23.5) | 56 (32.0) | 42 (25.0) | 45 (21.3) | 53 (22.5) | 76 (22.5) | 18 (22.2) | 69 (23.0) | 19 (19.4) | 0.24 |
| Guidelines, n (%) |  |  |  |  |  |  |  |  |  |  |
| ≥ 150 MVPA min per week | 1047 (65.2) | 123 (70.3) | 103 (61.3) | 134 (63.5) | 170 (72.0) | 213 (63.0) | 52 (64.2) | 185 (61.7) | 67 (68.4) | 0.14 |
| ≥ 2x BMSA min per week | 957 (59.6) | 124 (70.9) | 95 (56.5) | 117 (55.5) | 144 (61.0) | 201 (59.5) | 43 (53.1) | 175 (58.3) | 58 (59.2) | 0.07 |
| Both | 736 (45.8) | 99 (56.6) | 70 (41.7) | 89 (42.2) | 116 (49.2) | 147 (43.5) | 35 (43.2) | 128 (42.7) | 52 (53.1) | 0.05 |
| Do you have a balanced eating pattern? *Yes* | 1414 (88.0) | 159 (90.9) | 148 (88.1) | 179 (84.8) | 197 (83.5) | 309 (91.4) | 74 (91.4) | 265 (88.3) | 83 (84.7) | 0.06 |
| Do you eat an average of 2 pieces of fruit per day? *Yes* | 906 (56.4) | 107 (61.1) | 79 (47.0) | 125 (59.2) | 141 (59.7) | 196 (58.0) | 45 (55.6) | 155 (51.7) | 58 (59.2) | 0.28 |
| How many days a week do you eat vegetables? | 6.5 (0.8) | 6.4 (0.9) | 6.6 (0.8) | 6.4 (0.9) | 6.4 (0.9) | 6.6 (0.7) | 6.7 (0.6) | 6.6 (0.8) | 6.4 (0.9) | <0.01 |
| Have you been treated by a dietitian? *Yes* | 209 (13.0) | 32 (18.3) | 18 (10.7) | 31 (14.7) | 32 (13.6) | 51 (15.1) | 8 (9.9) | 23 (7.7) | 14 (14.3) | 0.03 |
| How satisfied are you with your sleep? (0-10) | 7.0 (1.6) | 7.0 (1.7) | 7.1 (1.7) | 7.0 (1.5) | 6.8 (1.7) | 6.9 (1.5) | 7.1 (1.5) | 7.1 (1.6) | 7.1 (1.7) | 0.65 |
| How much stress do you experience at work? (0-10) | 5.5 (2.0) | 5.6 (1.8) | 5.6 (1.9) | 4.9 (2.0) | 5.7 (1.9) | 5.5 (1.8) | 6.1 (1.9) | 5.7 (2.1) | 4.8 (2.1) | <0.001 |
| How much stress do you experience at home? (0-10) | 3.9 (2.2) | 3.8 (2.1) | 3.7 (2.2) | 3.2 (2.2) | 3.7 (2.1) | 4.1 (2.1) | 4.5 (2.2) | 4.1 (2.3) | 3.9 (2.2) | 0.01 |
| How important do you find your own lifestyle? (0-10) | 8.0 (1.1) | 8.3 (1.1) | 7.9 (1.1) | 8.2 (1.0) | 8.0 (2.0) | 8.0 (1.1) | 8.1 (1.1) | 8.0 (1.2) | 8.0 (1.3) | 0.12 |
| How fit do you consider yourself? (0-10) | 6.8 (1.4) | 7.0 (1.1) | 6.6 (1.4) | 6.8 (1.3) | 6.7 (1.4) | 6.7 (1.3) | 6.9 (1.4) | 6.9 (1.3) | 6.5 (1.7) | 0.01 |
| **Counselling and referring practices** | | | | | | | | | | |
| How important do you find lifestyle in psychiatric treatment? (0-10) | 8.1 (1.3) | 8.1 (1.2) | 7.9 (1.1) | 8.3 (1.4) | 8.4 (1.3) | 8.0 (1.2) | 8.0 (1.2) | 7.8 (1.4) | 8.6 (1.2) | <0.001 |
| Statement: lifestyle should be part of any psychiatric treatment |  |  |  |  |  |  |  |  |  | <0.001 |
| Totally disagree | 18 (1.1) | 2 (1.2) | 2 (1.2) | 2 (1.0) | 4 (1.7) | 3 (0.9) | 0 (0.0) | 3 (1.0) | 2 (2.1) |  |
| Disagree | 43 (2.7) | 1 (0.6) | 2 (1.2) | 4 (1.9) | 6 (2.6) | 18 (5.4) | 3 (3.7) | 6 (2.0) | 3 (3.1) |  |
| Neutral | 135 (8.5) | 22 (6.6) | 17 (10.2) | 14 (6.8) | 13 (5.6) | 22 (6.6) | 15 (18.5) | 39 (13.2) | 2 (2.1) |  |
| Agree | 807 (50.8) | 206 (61.5) | 91 (54.8) | 95 (45.9) | 94 (40.2) | 206 (61.5) | 36 (44.4) | 159 (53.7) | 34 (35.4) |  |
| Totally agree | 585 (36.8) | 86 (25.7) | 54 (32.5) | 92 (44.4) | 117 (50.0) | 86 (25.7) | 27 (33.3) | 89 (30.1) | 55 (57.3) |  |
| Who do you think is responsible for the lifestyle treatment of the psychiatric patient? |  |  |  |  |  |  |  |  |  |  |
| General practitioner | 700 (43.6) | 86 (49.1) | 113 (67.3) | 70 (33.2) | 93 (39.4) | 139 (41.1) | 35 (43.2) | 125 (41.7) | 39 (39.8) | <0.001 |
| Main psychiatric HCP | 414 (25.8) | 37 (21.1) | 68 (40.5) | 49 (23.2) | 69 (29.2) | 70 (20.7) | 19 (23.5) | 67 (22.3) | 35 (35.7) | <0.001 |
| Every psychiatric HCP | 1238 (77.0) | 133 (76.0) | 130 (77.4) | 156 (73.9) | 196 (83.1) | 245 (72.5) | 62 (76.5) | 231 (77.0) | 85 (86.7) | 0.03 |
| Caretakers or nurses | 699 (43.5) | 84 (48.0) | 101 (60.1) | 73 (34.6) | 110 (46.6) | 133 (39.3) | 27 (33.3) | 109 (36.3) | 62 (63.3) | <0.001 |
| Patient | 1045 (65.0) | 125 (71.4) | 142 (84.5) | 138 (65.4) | 134 (56.8) | 212 (62.7) | 50 (61.7) | 177 (59.0) | 67 (68.4) | <0.001 |
| Other | 104 (6.5) | 6 (3.4) | 10 (6.0) | 13 (6.2) | 17 (7.2) | 26 (7.7) | 7 (8.6) | 22 (7.3) | 3 (3.1) | 0.46 |
| What proportion of your patients do you ask about: |  |  |  |  |  |  |  |  |  |  |
| Smoking habits | 69.1 (32.1) | 60.8 (37.9) | 80.8 (18.5) | 55.0 (33.6) | 80.3 (25.4) | 62.3 (35.4) | 65.8 (32.4) | 79.2 (24.1) | 59.9 (35.7) | <0.001 |
| Drinking habits | 77.6 (25.9) | 71.8 (31.9) | 83.4 (16.0) | 70.7 (25.2) | 86.2 (18.8) | 73.1 (29.8) | 79.3 (23.9) | 85.8 (16.3) | 59.5 (36.1) | <0.001 |
| Physical activity habits | 58.2 (28.4) | 54.1 (30.5) | 37.9 (22.7) | 75.7 (19.4) | 70.4 (23.6) | 57.0 (28.7) | 53.4 (27.6) | 54.2 (27.7) | 53.3 (30.8) | <0.001 |
| Their eating pattern | 55.1 (28.5) | 54.9 (31.0) | 38.4 (25.1) | 58.9 (25.1) | 67.8 (24.9) | 57.3 (28.1) | 56.5 (28.8) | 49.4 (27.9) | 52.9 (30.6) | <0.001 |
| Their sleeping pattern | 84.0 (16.0) | 81.2 (18.4) | 85.4 (12.5) | 85.9 (12.1) | 87.9 (12.1) | 82.6 (16.8) | 80.7 (17.6) | 86.7 (12.5) | 71.8 (26.8) | <0.001 |
| What proportion of your patients do you ask about: |  |  |  |  |  |  |  |  |  |  |
| Physical activity habits | 58.2 (28.4) | 54.1 (30.5) | 37.9 (22.7) | 75.7 (19.4) | 70.4 (23.6) | 57.0 (28.7) | 53.4 (27.6) | 54.2 (27.7) | 53.3 (30.8) | <0.001 |
| Their dietary habits | 55.1 (28.5) | 54.9 (31.0) | 38.4 (25.1) | 58.9 (25.1) | 67.8 (24.9) | 57.3 (28.1) | 56.5 (28.8) | 49.4 (27.9) | 52.9 (30.6) | <0.001 |
| Their sleeping pattern | 84.0 (16.0) | 81.2 (18.4) | 85.4 (12.5) | 85.9 (12.1) | 87.9 (12.1) | 82.6 (16.8) | 80.67 (17.6) | 86.7 (12.5) | 71.8 (26.8) | <0.001 |
| **Only MHCPs of adult patients** |  |  |  |  |  |  |  |  |  |  |
| Tobacco use | 72.6 (29.8) | 69.1 (34.5) | 80.9 (18.5) | 55.1 (33.7) | 81.7 (24.3) | 69.6 (32.3) | 67.6 (30.9) | 79.9 (23.4) | 67.8 (32.2) | <0.001 |
| Alcohol use | 81.8 (21.2) | 81.6 (23.1) | 84.0 (15.5) | 71.0 (24.9) | 87.6 (16.7) | 82.6 (21.0) | 82.0 (21.0) | 87.3 (21.2) | 67.1 (33.7) | <0.001 |
| What proportion of your patients who smoke do you advise to stop smoking? | 43.0 (32.2) | 27.2 (29.0) | 48.3 (28.1) | 42.1 (31.6) | 56.1 (31.2) | 32.4 (30.5) | 33.8 (30.7) | 54.9 (30.6) | 41.3 (33.4) | <0.001 |
| **Only MHCPs of adult patients**  What proportion of your patients who smoke do you advise to stop smoking? | 44.2 (31.3) | 27.9 (27.6) | 47.4 (27.8) | 41.9 (31.9) | 54.9 (30.9) | 33.3 (28.5) | 35.0 (30.3) | 54.1 (30.1) | 45.6 (32.7) | <0.001 |
| What proportion of your patients do you refer to a lifestyle intervention to improve their mental health? | 41.1 (27.6) | - | 35.2 (25.1) | 47.6 (27.3) | 48.1 (27.7) | 35.8 (26.9) | 34.9 (27.6) | 41.9 (28.0) | - | <0.001 |
| Where do you refer to? |  |  |  |  |  |  |  |  |  |  |
| Psychomotor therapist | 531 (39.8) | - | 91 (54.2) | 56 (26.5) | 83 (35.2) | 143 (42.3) | 34 (42.0) | 124 (41.3) | - | <0.001 |
| Physiotherapy | 444 (33.3) | - | 56 (33.3) | 115 (54.5) | 64 (27.1) | 101 (29.9) | 21 (25.9) | 87 (29.0) | - | <0.001 |
| Physical activity worker? | 102 (7.6) | - | 18 (10.7) | 15 (7.1) | 32 (13.6) | 14 (4.1) | 4 (4.9) | 19 (6.3) | - | <0.001 |
| Regular sports activities | 642 (48.1) | - | 51 (30.4) | 134 (63.5) | 131 (55.5) | 157 (46.4) | 33 (40.7) | 136 (45.3) | - | <0.001 |
| Dietician | 594 (44.5) | - | 79 (47.0) | 91 (43.1) | 106 (44.9) | 134 (39.6) | 34 (42.0) | 150 (50.0) | - | 0.17 |
| Other | 307 (23.0) | - | 21 (12.5) | 60 (28.4) | 68 (28.8) | 71 (21.0) | 23 (28.4) | 64 (21.3) | - | <0.001 |
| What proportion of your patients do you refer to a lifestyle intervention to improve their psychical health? | 37.9 (26.7) | - | 30.9 (22.7) | 42.7 (27.4) | 46.7 (27.2) | 32.4 (25.3) | 35.4 (27.6) | 38.4 (26.5) | - | <0.001 |
| Where do you refer to? |  |  |  |  |  |  |  |  |  |  |
| Psychomotor therapist | 331 (24.8) | - | 53 (31.5) | 36 (17.1) | 61 (25.8) | 81 (24.0) | 25 (30.9) | 75 (25.0) | - | 0.03 |
| Physiotherapy | 512 (38.4) | - | 60 (35.7) | 108 (51.2) | 91 (38.6) | 125 (37.0) | 27 (33.3) | 101 (33.7) | - | <0.01 |
| Physical activity worker | 101 (7.6) | - | 17 (10.1) | 16 (7.6) | 25 (10.6) | 18 (5.3) | 4 (4.9) | 21 (7.0) | - | 0.16 |
| Regular sports activities | 687 (51.5) | - | 62 (36.9) | 127 (60.2) | 137 (58.1) | 172 (50.9) | 39 (48.1) | 150 (50.0) | - | <0.001 |
| Dietician | 558 (41.8) | - | 77 (45.8) | 82 (38.9) | 99 (41.9) | 128 (37.9) | 27 (33.3) | 145 (48.3) | - | 0.04 |
| Other | 253 (19.0) | - | 13 (7.7) | 44 (20.9) | 67 (28.4) | 53 (15.7) | 18 (22.2) | 58 (19.3) | - | <0.001 |
| What proportion of your patients with sleeping problems do you refer to insomnia CBT/do you give insomnia CBT yourself? | 29.7 (25.8) | - | 25.3 (24.2) | 37.8 (27.7) | 31.2 (28.1) | 26.4 (24.1) | 32.3 (25.1) | 28.4 (24.3) | - | <0.001 |
| **Table S1. Results of the questionnaire, divided in profession groups.** BMI=body mass index, BMSA=bone and muscle strengthening activities, CBT=cognitive behavioral therapy, GP=general practitioner, MHCP=mental health care professional, MVPA=moderate to vigorous physical activity, n=number, PA=physical activity, SD=standard deviation. | | | | | | | | | | |

| **Variable** | **Take history on** | | | | | | | | | | | | |
| --- | --- | --- | --- | --- | --- | --- | --- | --- | --- | --- | --- | --- | --- |
|  | **Physical activity n=1579** | | | **Dietary habits n=1578** | | **Sleep n=1575** | | | | **Tobacco use n=1274** | | **Alcohol use n=1279** | |
|  | **OR** | **CI** | | **OR** | **CI** | **OR** | | **CI** | | **OR** | **CI** | **OR** | **CI** |
| Age per 10 years | 1.13 | 1.03 - 1.24 | | 0.99 | 0.90 - 1.09 | 0.92 | | 0.83 - 1.02 | | 1.07 | 0.96 - 1.20 | 1.08 | 0.96 - 1.21 |
| Female | 1.31 | 1.05 - 1.63 | | 1.61** | 1.29 - 2.02 | 1.66** | | 1.31 - 2.11 | | 1.22 | 0.95 - 1.57 | 1.19 | 0.91 - 1.55 |
| BMI per 5 units | 0.99 | 0.86 - 1.13 | | 1.02 | 0.89 - 1.16 | 1.13 | | 0.98 - 1.31 | | 1.09 | 0.93 - 1.28 | 1.08 | 0.92 - 1.27 |
| How important own lifestyle | 1.18** | 1.08 - 1.29 | | 1.18** | 1.08 - 1.29 | 1.26** | | 1.15 - 1.39 | | 1.18** | 1.06 - 1.31 | 1.13 | 1.02 - 1.26 |
| Smoking (reference = smoker) | | | | | | | | | | | | | |
| *Smoked in past* | 1.59 | 1.00 - 2.53 | | 1.27 | 0.81 - 1.99 | 1.01 | | 0.60 - 1.69 | | 1.28 | 0.76 - 2.16 | 0.94 | 0.53 - 1.69 |
| *Never smoked* | 1.50 | 0.99 - 2.26 | | 1.12 | 0.75 - 1.68 | 0.75 | | 0.47 - 1.19 | | 1.10 | 0.69 - 1.77 | 0.73 | 0.43 - 1.25 |
| Doesn't drink alcohol | 0.86 | 0.71 - 1.05 | | 0.91 | 0.75 - 1.11 | 1.02 | | 0.82 - 1.26 | | 1.08 | 0.85 - 1.36 | 1.08 | 0.85 - 1.39 |
| Physical activity norm (reference = meets non criteria) | | | | | | | | | | | | | |
| *Meets 2 criteria* | 1.44** | 1.14 - 1.83 | | 1.38* | 1.09 - 1.75 | 1.08 | | 0.84 - 1.40 | | 1.47* | 1.11 - 1.93 | 1.64** | 1.23 - 2.18 |
| *Meets 1 criterium* | 1.42** | 1.11 - 1.81 | | 1.26 | 0.99 - 1.61 | 1.00 | | 0.77 - 1.30 | | 1.42 | 1.07 - 1.89 | 1.41 | 1.05 - 1.89 |
| Has a balanced eating pattern | 1.37 | 1.04 - 1.80 | | 1.18 | 0.90 - 1.56 | 0.92 | | 0.68 - 1.24 | | 0.95 | 0.69 - 1.31 | 1.11 | 0.79 - 1.56 |
| Sleep | 1.03 | 0.97 - 1.09 | | 1.00 | 0.95 - 1.06 | 1.09** | | 1.03 - 1.16 | | 1.05 | 0.99 - 1.12 | 1.07 | 1.00 - 1.14 |
| Profession (reference = psychiatrist) | | | | | | | | | | | | | |
| *Other* | 1.00 | 0.65 - 1.54 | | 1.06 | 0.69 - 1.64 | 0.22* | | 0.13 - 0.35 | | 0.50 | 0.30 - 0.86 | 0.22** | 0.13 - 0.39 |
| *Psychologist* | 1.04 | 0.71 - 1.52 | | 1.16 | 0.80 - 1.69 | 0.44* | | 0.30 - 0.66 | | 0.60 | 0.39 - 0.94 | 0.73 | 0.46 - 1.16 |
| *Resident in psychiatry* | 0.46** | 0.32 - 0.66 | | 0.52** | 0.36 - 0.74 | 0.72 | | 0.49 - 1.07 | | 1.02 | 0.68 - 1.52 | 0.70 | 0.46 - 1.07 |
| *GP based nurse specialist* | 3.58** | 2.60 - 4.93 | | 1.54* | 1.13 - 2.11 | 0.69* | | 0.49 - 0.97 | | 0.21** | 0.14 - 0.29 | 0.21** | 0.14 - 0.30 |
| *Referring MHCP with nursing background* | 2.88** | 2.11 - 3.93 | | 2.94** | 2.16 - 4.01 | 1.17 | | 0.83 - 1.67 | | 1.32 | 0.92 - 1.90 | 1.37 | 0.92 - 2.05 |
| *Referring MHCP with scientific background* | 1.24 | 0.92 - 1.66 | | 1.47* | 1.10 - 1.96 | 0.56** | | 0.41 - 0.77 | | 0.61 | 0.42 - 0.87 | 0.75 | 0.51 - 1.09 |
| *Clinical psychologist* | 0.92 | 0.60 - 1.41 | | 1.50 | 0.97 - 2.33 | 0.43** | | 0.27 - 0.67 | | 0.45** | 0.27 - 0.73 | 0.62 | 0.37 - 1.05 |
| *Stress at work* | 0.98 | 0.94 - 1.03 | | 1.00 | 0.96 - 1.05 | 1.01 | | 0.96 - 1.07 | | 0.99 | 0.93 - 1.04 | 1.01 | 0.95 - 1.06 |
|  | **Advice to quit smoking n=1090** | | **Referral to improve mental health n=1305** | | | | **Referral to improve physical health n=1295** | | | **Referral to insomnia CBT n=1294** | |  |  |
| **Variable** | OR | CI | OR | | CI | | OR | | CI | OR | CI |  |  |
| Age per 10 years | 0.97 | 0.87 - 1.09 | 1.09 | | 0.98 - 1.20 | | 1.07 | | 0.96 - 1.18 | 1.06 | 0.95 - 1.18 |  |  |
| Female | 0.87 | 0.68 - 1.11 | 1.36 | | 1.08 - 1.73 | | 1.18 | | 0.93 - 1.50 | 1.44** | 1.13 - 1.83 |  |  |
| BMI per 5 units | 0.94 | 0.80 - 1.12 | 0.90 | | 0.78 - 1.04 | | 0.90 | | 0.78 - 1.04 | 0.91 | 0.79 - 1.05 |  |  |
| How important own lifestyle | 1.15 | 1.04 - 1.28 | 1.18** | | 1.07 - 1.29 | | 1.141* | | 1.04 - 1.26 | 1.02 | 0.92 - 1.12 |  |  |
| Smoking (reference = smoker) | | | | | | | | | | | |  |  |
| *Smoked in past* | 3.30** | 1.89 - 5.76 | 0.67 | | 0.39 - 1.15 | | 0.60 | | 0.35 - 1.03 | 0.92 | 0.53 - 1.58 |  |  |
| *Never smoked* | 2.57** | 1.55 - 4.28 | 0.74 | | 0.55 - 0.98 | | 0.87 | | 0.65 - 1.15 | 1.11 | 0.83 - 1.49 |  |  |
| Doesn't drink alcohol | 1.21 | 0.95 - 1.54 | 0.84 | | 0.68 - 1.05 | | 0.84 | | 0.67 - 1.04 | 1.07 | 0.86 - 1.34 |  |  |
| Physical activity norm (reference = meets non criteria) | | | | | | | | | | | |  |  |
| *Meets 2 criteria* | 1.57** | 1.18 - 2.09 | 1.30 | | 1.00 - 1.69 | | 1.46* | | 1.12 - 1.90 | 1.33 | 1.02 - 1.73 |  |  |
| *Meets 1 criterium* | 1.55** | 1.16 - 2.09 | 1.36 | | 1.04 - 1.79 | | 1.48* | | 1.13 - 1.94 | 1.19 | 0.91 - 1.55 |  |  |
| Has a balanced eating pattern | 0.87 | 0.62 - 1.20 | 1.00 | | 0.73 - 1.35 | | 0.95 | | 0.70 - 1.29 | 1.09 | 0.80 - 1.49 |  |  |
| Sleep | 1.02 | 0.95 - 1.09 | 1.08 | | 1.01 - 1.14 | | 1.02 | | 0.96 - 1.09 | 1.03 | 0.97 - 1.10 |  |  |
| Profession (reference = psychiatrist) | | | | | | | | | | | |  |  |
| *Resident in psychiatry* | 0.72 | 0.49 - 1.07 | 0.74 | | 0.51 - 1.07 | | 0.679 | | 0.47 - 0.99 | 0.79 | 0.54 - 1.15 |  |  |
| *GP based nurse specialist* | 0.50** | 0.35 - 0.70 | 1.40 | | 1.01 - 1.93 | | 1.31 | | 0.95 - 1.82 | 1.77** | 1.27 - 2.46 |  |  |
| *Referring MHCP with nursing background* | 1.13 | 0.81 - 1.58 | 1.50 | | 1.09 - 2.04 | | 1.77** | | 1.30 - 2.43 | 1.07 | 0.78 - 1.47 |  |  |
| *Referring MHCP with scientific background* | 0.31** | 0.22 - 0.44 | 0.67* | | 0.50 - 0.91 | | 0.67* | | 0.50 - 0.90 | 0.81 | 0.60 - 1.09 |  |  |
| *Clinical psychologist* | 0.31** | 0.19 - 0.52 | 0.58 | | 0.37 - 0.91 | | 0.77 | | 0.49 - 1.20 | 1.29 | 0.85 - 1.97 |  |  |
| *Stress at work* | 1.06 | 1.00 - 1.12 | 1.04 | | 0.99 - 1.10 | | 1.05 | | 0.99 - 1.10 | 1.04 | 0.99 - 1.09 |  |  |
| **Table S2. Results of the ordinal regression sensitivity analyses on lifestyle habits, gender, profession and lifestyle history and referral practices.** *=p<0.01, **=p<0.006. CI= confidence interval, GP=general practitioner, MHCP=health care professional, n=number, OR= odds ratio | | | | | | | | | | | |  |  |
